# Fused in Sarcoma (FUS) in DNA Repair: Tango with Poly(ADP-ribose) Polymerase 1 and Compartmentalisation of Damaged DNA

**DOI:** 10.3390/ijms21197020

**Published:** 2020-09-24

**Authors:** Maria V. Sukhanova, Anastasia S. Singatulina, David Pastré, Olga I. Lavrik

**Affiliations:** 1 Institute of Chemical Biology and Fundamental Medicine, SB RAS, 630090 Novosibirsk, Russia; lasty@ngs.ru (A.S.S.); lavrik@niboch.nsc.ru (O.I.L.); 2 Laboratoire Structure-Activité des Biomolécules Normales et Pathologiques, INSERM U1204, Université Paris-Saclay, 91025 Evry, France; david.pastre@univ-evry.fr

**Keywords:** fused in sarcoma, DNA repair, poly(ADP-ribose) polymerase, poly(ADP-ribose), protein phase separation

## Abstract

The fused in sarcoma (FUS) protein combines prion-like properties with a multifunctional DNA/RNA-binding domain and has functions spanning the regulation of RNA metabolism, including transcription, pre-mRNA splicing, mRNA transport and translation. In addition to its roles in RNA metabolism, FUS is implicated in the maintenance of DNA integrity. In this review, we examine the participation of FUS in major DNA repair pathways, focusing on DNA repair associated with poly(ADP-ribosyl)ation events and on how the interaction of FUS with poly(ADP-ribose) may orchestrate transient compartmentalisation of DNA strand breaks. Unravelling how prion-like RNA-binding proteins control DNA repair pathways will deepen our understanding of the pathogenesis of some neurological diseases and cancer as well as provide the basis for the development of relevant innovative therapeutic technologies. This knowledge may also extend the range of applications of poly(ADP-ribose) polymerase inhibitors to the treatment of neurodegenerative diseases related to RNA-binding proteins in the cell, e.g., amyotrophic lateral sclerosis and frontotemporal lobar degeneration.

## 1. Introduction

Throughout their lifespan organisms are constantly exposed to genotoxic agents, both exogenous and endogenous. A rough assessment has revealed that up to 70,000 DNA damage events occur per human cell per day [1]. Under these conditions, preserving cell genome integrity is one of the most important challenges faced by multicellular organisms. Notably, unrepaired DNA damage contributes to the appearance of a pathological mutation [2,3,4]. The maintenance of genome stability is achieved by the machineries associated with the DNA damage response (DDR) and the cell death pathways that carry out detection of DNA damage or a signal of its presence to orchestrate DNA repair and induce cell death upon massive DNA damage, respectively [5,6]. For a long time, it has been believed that RNA-binding proteins (RBPs) interfere indirectly with the DDR through post-transcriptional regulation of gene expression [7,8,9,10]. Nonetheless, more and more studies are revealing the direct functions of many RBPs in the presence of DNA damage, e.g., sensing, signalling, and repair [11,12,13]. In this regard, poly(ADP-ribose) polymerases (PARPs), which are DNA damage sensors, may provide the basis for the control of RBPs over DNA repair and accordingly aroused special interest recently [14,15,16,17,18,19]. Indeed, DNA damage followed by PARPs’ activation is accompanied with protein poly(ADP-ribosyl)ation (PARylation) through covalent attachment of ADP-ribose moieties and the formation of an ADP-ribose chain: poly(ADP-ribose) (PAR) [20,21,22]. PAR is a polymeric molecule that shares several features with RNA, including a high negative charge and structural diversity (size, chain length, and branching complexity) [23,24,25]. A large body of evidence suggests that many RBPs interact with PAR and/or undergo post-translational modification through PARylation during genotoxic stress [26,27]. Moreover, RBPs contain low-complexity regions with self-adhesive properties abundantly as compared to other proteins. Low-complexity domains (LCDs), also called intrinsically disordered regions, are considered key components of membrane-less assemblies such as DNA damage foci, P-bodies or stress granules formed under stressful conditions in the cell [28,29,30,31]. PAR, in turn, may serve as a scaffolding factor for these RBP-containing assemblies [16]. Just as many RBPs, fused in sarcoma (FUS) combines self-adhesive LCDs with multifunctional DNA/RNA-binding domains. FUS is involved in the regulation of RNA metabolism, including transcription, pre-mRNA splicing, mRNA transport and translation [32]. In addition to its role in RNA metabolism, FUS was recently implicated in the maintenance of DNA integrity in response to DNA damage [33,34]. In particular, FUS is mainly a nuclear protein [35] that interacts with DNA repair factors and is associated with DNA damage–induced formation of nuclear foci [17,36,37,38,39,40]. Nonetheless, the exact functions of FUS in DNA repair remain unclear. In this review, we highlight the role of FUS in the maintenance of genome integrity, focusing on PARylation events and on how the FUS–PAR interaction may be connected with DNA repair.

## 2. Intrinsically Disordered Regions and Prion-Like Properties of FUS

**Figure 1:**
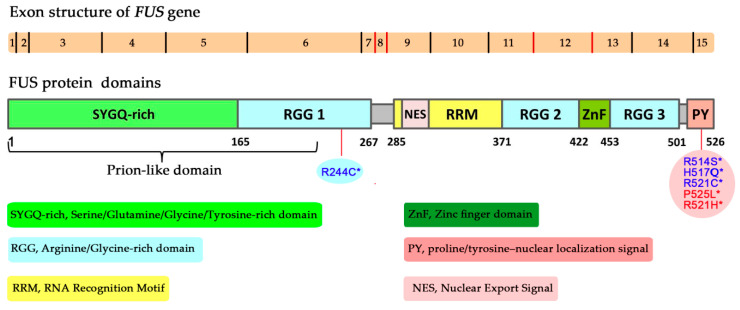
A schematic diagram of exon structure of the *FUS* gene and domain structure of the FUS protein [54]. * Mutations identified in patients with familial amyotrophic lateral sclerosis (fALS) and implicated in DNA repair and DDR [37,40].

The protein called fused in sarcoma (FUS; also known as translocated in liposarcoma; TLS) was first identified in human myxoid liposarcomas ~30 years ago, suggesting that this protein plays a critical part in cancers [41,42]. In liposarcoma, a chromosomal translocation leads to the fusion of two genes, *FUS* and UPR-regulated CCAAT/enhancer-binding protein homologous protein, resulting in the synthesis of a chimeric protein that acts as a transcription factor enhancing cell proliferation and promoting tumour progression [41]. FUS, along with Ewing’s sarcoma (EWS) and TATA-binding protein–associated factor 15 (TAF15), belongs to the FET family of RBPs that are highly conserved and perform functions primarily related to RNA metabolism [43,44]. FUS is a 526-amino-acid-long protein that possesses a serine/tyrosine/glycine/glutamine (SYGQ)-rich N terminus of low complexity, three arginine/glycine/glycine (RGG)-rich regions (named RGG1–3), a conserved RNA recognition motif, a zinc finger motif and a proline-tyrosine nuclear localisation signal at the C-terminus [45,46] (Figure 1). The C-terminal domains of FUS comprising the RNA recognition motif, RGG and zinc finger motifs mainly participate in the binding of FUS to RNA, DNA and PAR [47,48,49,50,51]. On the other hand, FUS’s unstructured N-terminal domain of low complexity is mainly associated with FUS self-interactions caused by homotypic multivalent interactions [52,53].

## 3. Higher-Order Assembly and Phase Separation of the FUS Protein for the Formation of Membrane-Less Assemblies in the Cell

**Figure 2:**
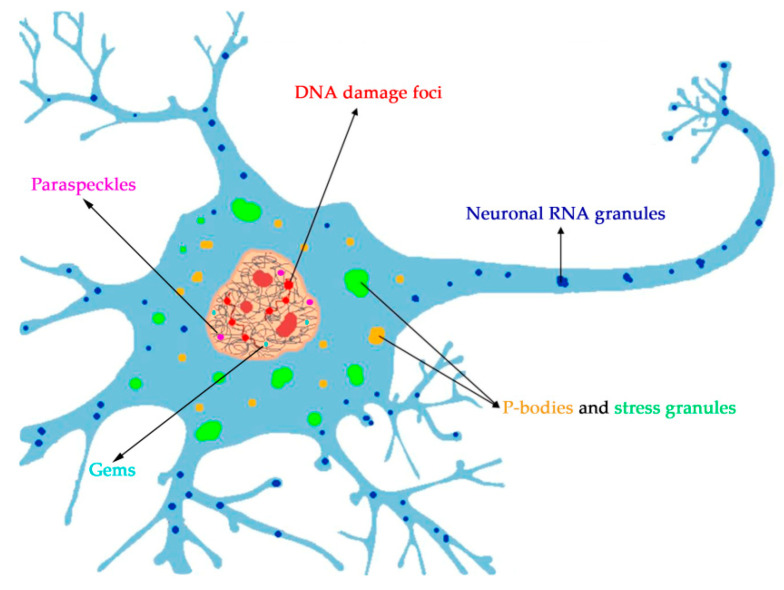
Schematic illustration of a neuronal cell and membrane-less compartments generated with the participation of FUS in the nucleus and cytoplasm. We corrected the Figure 2 (Please, see the attached file).

Notably, through its long N-terminal LCD with prion-like properties, higher-order multimolecular assembly of FUS either alone or in the presence of RNA gives rise to diverse structures including aggregates, hydrogels, amyloid fibrils and liquid droplets in vivo that have been the subject of intense research since pathological mutations in *FUS* were directly associated with two major neurodegenerative diseases, amyotrophic lateral sclerosis (ALS) and frontotemporal lobar degeneration (FTLD), and the identification of cytoplasmic inclusions of FUS in neurons of the affected patients [41,52,53,55,56,57]. In agreement with its ability to aggregate, among more than 200 yeast-prion-like proteins that have been identified in the human proteome [58], FUS has been ranked 15^th^ for its prion-like properties and 1st among RBPs [59]. In particular, it has been shown that SYGQ- and glycine-rich regions at the N terminus of FUS have prion-like properties and accordingly play an important role in FUS aggregation. In line with this notion, a truncated FUS protein lacking the N terminus is not able to form droplets or aggregates both in vitro and in vivo [17,56,60,61,62,63,64]. Weak homotypic multivalent intermolecular interactions occur between N-terminal LCDs thereby resulting in FUS self-assembly into liquid-like dynamical compartments as a single component. Heterotypic interaction with other proteins and/or nucleic acids can lead to heterogeneous higher-order structures giving rise to higher complexity in terms of composition, shape and dynamics and most probably biological functions [52,55,64,65]. In this way, it has been suggested that phase transition, in particular liquid–liquid phase separation (LLPS) of protein or protein–nucleic acid mixtures underlies the emergence of membrane-less compartments such as nucleoli, Cajal bodies, gemini of Cajal bodies, Nuage bodies, speckles, paraspeckles, DNA damage foci, stress granules and P-bodies in the cell [28,29,30,66]. In the emerging field of phase separation biology, FUS has received even more attention since pathological mutations in the LCD that impair FUS were shown to trigger a phase transition from a reversible liquid-like droplet or gel-like state to irreversible solid-like states possibly promoting the formation of cytoplasmic inclusions of FUS found in ALS and FTLD [66]. Taking into account that FUS undergoes phase separation and interacts with other macromolecules such as RNA, DNA or PAR, FUS is regarded as an important player in the creation of membrane-less compartments in vivo under physiological and stressful conditions [67,68]. Indeed, nuclear FUS has been detected in association with DNA damage foci [17,37], paraspeckles [69] and SMN1 bodies (Gems) [70], whereas cytoplasmic FUS is recruited into stress granules or P-bodies [71,72,73,74,75,76,77,78,79,80] and neuronal RNA granules [81,82] (Figure 2).

Accordingly, the functions of FUS in DNA repair may be related to its capacity to induce the formation of dynamic compartments that regulate DNA repair through protein phase separation. Although numerous studies have shown that FUS can be recruited to a region containing DNA damage in the nucleus, whether FUS directly affects the efficiency of DNA repair, by promoting the emergence of DNA repair foci, is still an open question.

## 4. Direct Functions of FUS in DNA Repair

As mentioned above, FUS mainly features a nuclear localisation [83], while a smaller proportion of FUS is found in the cytoplasm under physiological conditions [48,82]. For a long time, FUS has been known to be involved in the regulation of RNA metabolism [43,84]. FUS binds preferentially to a nascent GU-rich mRNA transcript and has been identified as a component of membrane-less organelles associated with RNA processing such as SMN1 bodies (Gems) in the nucleus and RNA granules or stress granules in the cytoplasm [70].

**Figure 3:**
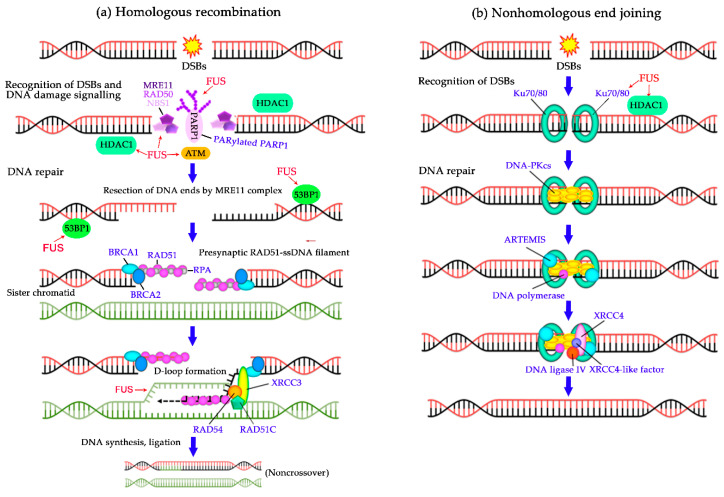
The involvement of FUS in double-strand DNA break (DSB) repair pathways. Schematic overview of HR, NHEJ illustrating basic steps of these pathways along with the proteins implicated in each step [86]. (**a**) The influence of FUS on HR. Simplified scheme for HR. The Mre11/Rad50/NBS1 complex starts resection on the DSBs to generate single stranded DNA. The ssDNA is first coated by Replication protein A (RPA), which is subsequently replaced by Rad51 with the help of BRCA1 and BRCA2. These Rad51 nucleoprotein filaments mediate strand invasion on the homologous template. The invading 3′-end of ssDNA serves as a primer for DNA synthesis. D-loop strands extended by DNA repair synthesis dissociate from their sister chromatid complements and reanneal with their original complementary strands. Additional DNA synthesis in the reannealed DNA duplex and ligation of the remaining single strand nicks complete the repair in the case of synthesis-dependent strand-annealing model of D-loop resolution, forming non-crossover products. FUS interacts with histone deacetylase 1 (HDAC1) and PARylated PARP1, binds to a D-loop intermediate and affects DSB-dependent accumulation of ATM (Ataxia-telangiectasia mutated serine-proteins kinase), NBS1 (Nijmegen breakage syndrome 1) and 53BP1 (p53-binding protein 1) [37,49]. (**b**) The effect of FUS on NHEJ. Simplified scheme for NHEJ. The two broken DNA ends are processed by the action of the end-binding Ku70/80 heterodimer complex, DNA-dependent protein kinase, catalytic subunits, (DNA-PKcs), artemis, DNA polymerase and ligated by DNA Ligase IV-XRCC4 complex. FUS interacts with HDAC1 and affects accumulation of Ku70/80 at DSBs [37].

**Figure 4:**
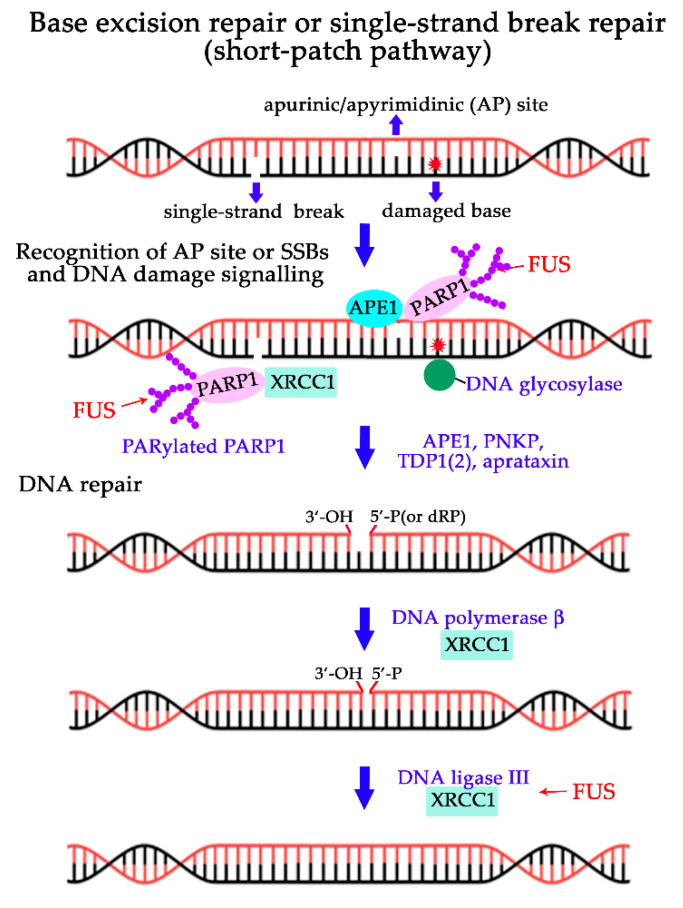
The involvement of FUS in base excision repair (BER) or single-strand break (SSB) repair pathways. Simplified scheme for BER/SSBR short-patch pathway [87]. Monofunctional DNA glycosylases catalyse the removal of the damaged base through cleavage of the *C1’-N*-glycosylic bond, leaving an AP site. AP endonuclease1 (APE1) cleaves AP site, DNA polymerase β inserts a single nucleotide and removes the 5′-deoxyribose phosphate (dRP), and the resulting nick is sealed by DNA ligase III-XRCC1 complex. In the case of SSB, the 5′-and 3′-termini containing blocking modifications can be converted to 5′-phosphate (P) and 3′-hydroxyl (OH) moieties by APE1, polynucleotide kinase 3’-phosphatase (PNKP), tyrosyl-DNA phosphodiesterase 1/2 (TDP1, TDP2) and/or aprataxin. AP sites, SSBs, arising endogenously or exogenously are bound by PARP1, which is then activated and autoPARylated. AutoPARylated PARP1 recruits repair proteins, in particular XRCC1 to the SSB [87]. The impact of FUS on BER is as follows: FUS interacts with PARylated PARP1 and the DNA ligase III–XRCC1 complex, thereby stimulating the ligation of DNA ends [40].

More recently, the recruitment of FUS to DNA damage–induced foci in the nucleus has drawn attention to its involvement in DNA repair processes [17,38,40,51]. DNA damage repair proceeds through one of five major pathways: direct repair of certain types of UV light–induced photo-lesions or methylated bases; homologous recombination (HR) or nonhomologous end-joining (NHEJ) to repair double-strand breaks (DSBs); nucleotide excision repair of bulky lesions; base excision repair (BER) of damaged bases, apurinic/apyrimidinic sites and single-strand breaks (SSBs); and mismatch repair of unpaired bases [85]. Numerous studies indicate that FUS’s functions in the DDR are associated with DNA strand break signalling and the repair of oxidative DNA damage types such as oxidised DNA bases and DNA SSBs and DSBs [33,34]; therefore, here we focus on the possible participation of FUS in HR, NHEJ or BER pathways (Figure 3 and Figure 4).

### 4.1. A FUS Gene Knockdown in Mice and Cells Impairs HR

One of the first pieces of evidence that FUS may play an important part in the maintenance of genome stability has been provided by experiments with FUS-deficient mice and cells originating from the knockout animals. The first *FUS* knockout mice were generated by disruption of a region (homologous to the 12th exon of the human gene) encoding a domain including the zinc finger motif (Figure 1), resulting in a lack of normal transcripts or protein expression in mice [88]. The homozygous mouse pups fail to suckle, and most of them die shortly, within 16 h, after birth. *FUS^–/–^* primary fibroblasts or B lymphocytes derived from the knockout mice are characterised by genomic instability, and the lymphocyte proliferative response to mitogens is significantly affected. Those authors have suggested that FUS acts as a modulator or effector of gene expression by binding to RNA and thereby participates indirectly in the cellular response to DNA damage or mitogenic stimuli [88]. Another strain of FUS-deficient mice has been created via disruption of a region homologous to the 8^th^ exon of the human gene [89] (Figure 1). These *FUS^–/–^* animals manifest complete male sterility and reduced fertility of females; moreover, these mice and their fibroblasts (*FUS^–/−^)* are sensitive to ionising radiation. Detailed analyses have revealed that the FUS deficiency causes a defect in homologous pairing and synapsis during HR, thus leading to degeneration of spermatocytes (Figure 3a). According to measurements of homologous DNA-pairing activities in cell extracts, the contribution of FUS to ATP-independent annealing of complementary single-stranded DNAs and D-loop formation in superhelical double-stranded DNA has uncovered its role in homologous pairing [49]. Therefore, FUS may contribute to meiotic HR through interaction with a D-loop intermediate (Figure 3a). Meiotic HR is a programmed event, but HR is also one of two major DSB repair pathways, and homologous DNA pairing is an essential step in the repair pathway [90]. Consequently, defects in the repair of DNA damage produced by ionising radiation in FUS knockout animals and cells may be explained by impaired DSB repair via HR.

### 4.2. Deficiency of FUS or Mutations of FUS Affect the Repair of DNA Strand Breaks

Because FUS deficiency increases genome instability in animal and cell models, FUS has been the subject of further research on its role in the repair processes. As mentioned above, eukaryotic cells have evolved two pathways to repair DSBs, namely, HR and NHEJ (Figure 3a,b) [86,90]. Other experimental pieces of direct evidence for the participation of FUS in DSB repair come from examination of the effects of a knockdown of FUS by a small interfering RNA or expression of fALS-associated mutant FUS versions (R244C, R514S, H517Q or R521C) in murine primary neuronal culture and/or human osteosarcoma U2OS cells [37]. Upon DNA damage, FUS depletion leads to a decrease in the level of H2AX phosphorylation, an impairment of DSB repair foci creation and deficient accumulation of DDR proteins such as p53-binding protein 1 (53BP1), Nijmegen breakage syndrome 1 (NBS1), phospho-ATM (ataxia telangiectasia mutated) and Ku70 (Figure 3a,b). The role of FUS in DSB repair is further underscored by results of FUS depletion: decreased HR and NHEJ efficiency and elevating the number of DNA damages in primary neuronal culture [37]. Besides, FUS directly associates with chromatin [91] and with a remodelling factor such as histone deacetylase 1 (HDAC1) [37]. HDAC1 plays an important part in the promotion of DNA DSB repair in post-mitotic neurons [92]. Furthermore, the expression of FUS mutants defective in their interaction with HDAC1 impairs both NHEJ and HR pathways in the U2OS^−GFP^ cell line [37]. These observations indicate an impaired capacity to repair a DNA DSB when expression levels of FUS are low or FUS is mutated in the cell. The functions of FUS in NHEJ and HR repair pathways are therefore clearly non-transcriptional.

In addition to its functions in DSB repair, FUS is involved in DNA SSB repair in HEK293 cells [40]. FUS co-immunoprecipitates with BER players such as XRCC1 and DNA ligase III (Lig III) and stimulates Lig III activity via direct interaction. Moreover, a CRISPR/Cas9-mediated knockout of FUS in HEK293 cells reduces the efficiency of SSB repair [40]. Defective DNA repair of oxidative DNA damage is observed in induced pluripotent stem cell lines derived from ALS patients carrying either the R521H or P525L mutation of FUS (Figure 1), which promotes the formation of cytosolic FUS aggregates at the expense of the pool of nuclear FUS [66,93].

These findings therefore define FUS as a novel participant of DNA break repair processes that plays an upstream role in DSB signalling; these data also point to a functional link to HR, NHEJ and BER/SSB repair via interaction with DNA intermediates and/or repair factors. In the case of DSB repair (Figure 3a,b), FUS facilitates the initial recruitment of DNA damage signalling proteins to DNA lesions and regulates HDAC’s chromatin-remodelling activity [37]. In the case of SSB repair (Figure 4), FUS interacts with the XRCC1–DNA ligase III complex and stimulates the SSB ligation step [40]. On the other hand, we cannot rule out the possibility that FUS has functions downstream of ATM or DNA-PK in response to DNA breaks. This is because FUS phosphorylation drives its translocation to the cytoplasm under genotoxic stress thereby possibly affecting FUS functions in RNA metabolism [36,39].

### 4.3. FUS Is Connected to DNA Strand Breaks by PARPs’ Signalling Activities

**Figure 5:**
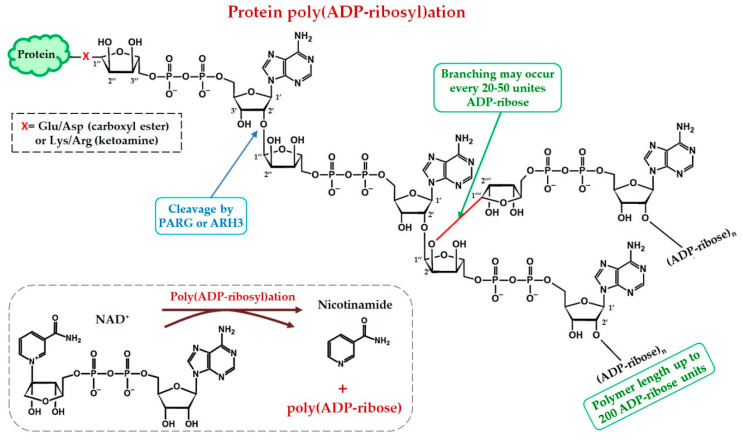
A diagram of the structure of PAR and protein PARylation. The scheme illustrates chemical structure of NAD^+^ and a chain of ADP-ribose units linked by the α(2′-1′′) *O*-glycosidic bond between ribose residues (linear chain) and by the α(2′′-1′′′) glycosidic bond between two nicotinamide-proximal ribose residues (branching). PARG and ARH3 is the main enzymes that degrade PAR and possesses exo- and endoglycosidase activities hydrolysing the glycosidic bonds between ribose units of PAR [98,99].

Although in vitro studies indicate that FUS directly binds to single- or double-stranded DNA or G-quadruplexes in telomeres [49,94], how FUS is recruited to the sites of DNA damage in the cell has been an open question. FUS recruitment to DNA repair sites has been analysed owing to the development of the laser micro-irradiation technique able to generate a spatially controlled DNA damage region in the cell nucleus [37,38,40]. The first evidence that FUS can be translocated directly to DNA damage foci was obtained in cells subjected to 405 nm laser micro-irradiation. Moreover, FUS recruitment to DSBs is specifically dependent on the activity of PARP1, thus pointing to an interaction between FUS and the PAR produced by PARP1 at DNA damage sites [51]. Later, PAR-dependent FUS accumulation at damaged-DNA sites has been detected in response to oxidative DNA damage induced by a UVA (320–400 nm) laser, suggesting the recruitment of FUS not only to DSBs but also to SSBs generated directly or indirectly during the repair of oxidised bases in DNA [38]. Therefore, DNA damage–dependent activation of PARPs and the synthesis of PAR are some of the ways in which FUS can be recruited to sites of DNA damage [37,38,40]. PARPs, primarily PARP1 and PARP2, can directly recognise damaged DNA, and their binding to it results in PARP activation [95,96]. PARP enzymes use NAD^+^ as a substrate and catalyse the transfer of an ADP-ribose residue from NAD^+^ to target protein-acceptors, leading finally to the synthesis of ADP-ribose chains (PAR) attached to proteins, mainly PARPs themselves [21] (Figure 5).

PAR chains is commonly linked to Lys/Arg or Glu/Asp residues attached to the C1” or C1”,C2”, C3” atoms of ADP-ribose with the formation of ketoamine and carboxyl ester, respectively (Figure 5). Moreover, amino acid specificity of PARP1 (PARP2) from Glu/Asp to Ser residue can be changed under influence of other protein factors such as histone PARylation factor 1 [95,97].

Protein PARylation is a reversible process mainly due to poly(ADP-ribose) glycohydrolase (PARG) activity and ADP-ribosyl-acceptor hydrolases 3 (ARH3), which catalyse the cleavage between ADP-ribose structural units at the terminal position and inside the polymer, thereby releasing ADP-ribose or oligo(ADP-ribose), respectively [98,99]. Thus, the action of PAR-degrading enzymes makes protein PARylation a dynamic and reversible post-translational modification and plays an important role in the regulation of DNA repair [98,99,100]. Protein PARylation, PAR length and formation of protein-free PAR molecules all contribute to the regulation of DNA repair, in particular, through the binding of proteins to PAR or their PARylation [16,21,25,100]. Therefore, PAR (either attached to proteins or free), because of its biochemical properties and tight regulation of its synthesis and degradation by PARP and PARG/ARH3 activities, is considered a critical factor that orchestrates reversible assembly of DNA repair compartments [16,25,99,101]. It is commonly accepted now that PARPs and PARylation regulate DSB and BER/SSB repair [102,103]. Recent research showed that FUS binds to PAR non-covalently and/or can be PARylated in vivo and in vitro [17,26,27,40,101]. Consistently with this notion, the FUS interaction with PAR after DNA damage constitutes the missing link between FUS and DNA repair events [17,38,40,51,56,101]. In line with this view, PAR synthesis at DNA damage sites induces the relocation of FUS to DNA damage foci [17,38,40,51]. Furthermore, inhibition of PARP activity impairs FUS accumulation at sites of laser-induced damage in the cell, and it is likely that the absence of PAR prevents FUS from being directed to DNA damage sites [17,38,51].

Consequently, FUS may take part in DNA strand break repair in a PAR-dependent manner, although little is known about the functional significance of FUS–PAR interactions in DNA repair [40,104]. One supposition is the unusual capacity of FUS to form a liquid-like compartment making it an ideal organiser of DNA repair compartments [17,101,104] able to concentrate damaged DNA at the early stages of the DNA strand break response in order to undergo spatially controlled PAR-dependent phase separation [101,104].

### 4.4. DNA Damage Sensing by the Phase Separation of FUS?

**Figure 6:**
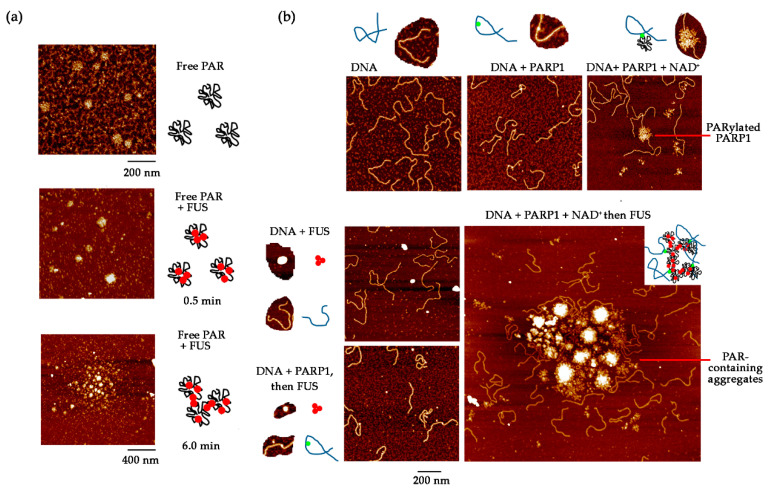
The formation of large DNA-rich assemblies in the presence of FUS after PAR synthesis by PARP1 [101]. (**a**) Representative AFM images of PAR:FUS complexes at different incubation times. PAR: 1 µM; FUS: 40 nM. (**b**) Representative AFM images of 1200-bp nicked DNA (1.25 nM) after incubation with PARP1 (3 nM) for 5 min in the presence or absence of NAD^+^ (0.3 mM) followed by the addition of FUS (40 nM) and incubation for 1 min.

Even though the nucleus, unlike the cytoplasm, does not contain membrane-bound organelles, the nucleus is a highly organised structure with separate proteinaceous and nucleic-acid–rich subnuclear compartments that have special morphology and specific composition of protein–nucleic acid complexes and are functionally specialised [105]. It was recently proposed that many DNA- or RNA-containing structures in the nucleus are assembled through a physicochemical process called LLPS [30,105,106,107]. In fact, LLPS of protein or protein–nucleic acid mixtures is now regarded as the principal mechanism behind the formation of protein-rich membrane-less compartments in the cell [29,105,106,107,108]. In eukaryotes, an abundant group of RBPs—that are intrinsically disordered proteins (IDPs) with prion-like properties—represents key factors that contribute to the creation of these compartments in the cell through the phase separation mechanism [30,64,65,67]. Among these proteins is FUS, which is presently intensively studied due to its ability to undergo reversible phase separation in vitro and in vivo (thereby generating protein-rich droplets, hydrogels and amyloid aggregates) and due to its link with major neurodegenerative diseases such as ALS [56,64,79,109,110]. Moreover, other biomolecules, such as RNA and PAR, influence the phase behaviour of FUS and can drive phase separation [17,56,101,111]. For example, functional interactions between RNA and FUS are assumed to play a key role in the dynamics of compartments among which we can find mRNA-rich stress granules [71,74,78,83,112]. The similarity to its association with RNA and the evidence of FUS condensation at a damaged-DNA site mediated by its interaction with PAR both in vitro and in cell systems have been reported [17,38,51,56,101]. *In vitro,* FUS has been found to interact with purified PAR directly, and its binding to PAR promotes LLPS of FUS and possibly its aggregation [16,17,38,56,101]. In addition, the appearance of FUS-rich assemblies in nuclear damage regions observed in vivo depends on PAR synthesised at DNA damage sites [17,56]. Accordingly, the interaction of FUS with PAR and the ensuing phase separation may be an important process underlying the formation of DNA repair compartments [17,56,101]. Nevertheless, the exact molecular mechanism(s) responsible for the creation of these compartments and their possible functions are difficult to address in a cellular context. At least three mechanisms have been considered to explain the formation of protein-rich DNA compartments in the nucleus, namely, (i) a cooperative binding of proteins to specific sites along DNA without phase separation, (ii) polymer–polymer phase separation in which proteins form molecular ‘bridges’ between different binding sites and (iii) LLPS of IDPs and nucleic acids [106,113]. So far, it is not clear what types of mechanisms take place in the context of a compartment generated to orchestrate DNA repair after FUS is directed to DNA damage sites upon PARP1 activation. Single-molecule experiments have partly shed light on this important issue. The atomic force microscopy single-molecule technique has been used to analyse the assemblies orchestrated by FUS at DNA damage sites after PARP1 activation in a reconstituted molecular system that includes mRNA to mimic mRNA targets of FUS in the nucleus, damaged DNA, PARP1 to recognise damage sites in DNA, NAD^+^ to trigger the synthesis of PAR by PARP1 and PARG to hydrolyse PAR [101]. Indeed, in the presence of free PAR, FUS can assemble into large PAR-containing aggregates (Figure 6a). Of note, when auto-PARylated PARP1 is still complexed with a DNA damage site, the FUS interaction with PAR gives rise to large aggregates in which DNA damage sites are condensed (Figure 6b).

**Figure 7:**
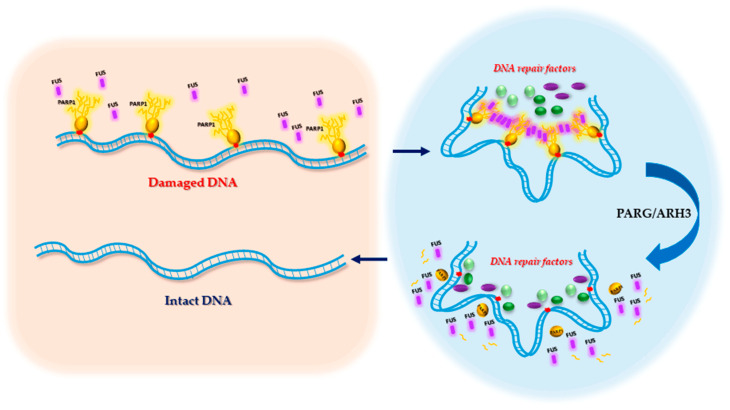
Creation of a repair compartment driven by the interaction of FUS with PAR. The compartment is a cluster of PARylated PARP1 bound by FUS that is concentrated to create a genomic region that is active in terms of DNA repair and may concentrate damaged DNA with subsequent recruitment of DNA repair proteins. PARG dissociates damaged DNA compartments by hydrolyzing PAR.

Thus, damaged DNA is rapidly confined to multi-protein complexes assembled after the FUS interaction with PARylated PARP1, thereby initiating the spatial segregation of damaged DNA (from intact DNA) into dynamical compartments [101]. The following sequence of events may then take place: (i) the site-specific interaction of PARP1 with damaged DNA triggers PAR synthesis to direct FUS to DNA damage sites, (ii) FUS induces the compartmentalisation of damaged DNA if PARylated PARP1 still bound to damaged DNA [101,114]. In agreement with the latter point, FUS fails to support the assembly of the DNA-rich compartments in mixtures of damaged DNA and free PAR [101]. Moreover, PAR hydrolysis by PARG induces the disassembly of DNA-rich compartments and a release of FUS. Due to the PARG/ARH3 activities, the formation of damaged-DNA–rich compartments by FUS is dynamic and reversible, which is important for the turnover of DNA repair. Taken together, these observations suggest that FUS-mediated DNA repair compartments form not only through the LLPS capacity of FUS but may also be due to mechanisms underlying the binding of PARP1 to damaged DNA and then bridging by FUS after PAR synthesis (Figure 7). Thus, PARP1 binding to damaged DNA proceeds without phase separation, then the interaction of FUS with PARylated PARPs bridges damage sites in close spatial proximity to each other.

Because some of the DNA repair factors interact with PAR [25,99,115,116,117], they can be in turn directed to the damaged-DNA–rich FUS/PAR compartments. Such a function in the organisation of DNA damage repair has previously been attributed to PAR itself [116,118], but the presence of an IDP like FUS may be necessary to increase the capacity of PAR to recruit DNA repair factors and/or to concentrate the DNA damage sites within the compartment. Consequently, the FUS interaction with PAR not only results in the emergence of damaged-DNA–rich compartments but also can stimulate the assembly of the relevant DNA machineries. Altogether, these findings provide new insights into DNA repair regulation at least for SSB repair and BER, because these processes are highly dependent on PARP1 activation [102,103]. The interaction of key proteins of BER/SSB repair pathway (Figure 4) such as AP endonuclease1, XRCC1 and DNA polymerase β with PAR was recently demonstrated [117]. FUS directly affects the efficiency of a repair process, for example, nick sealing by DNA ligase III at the last step of the repair pathways [40]. The spatiotemporal control of FUS/PAR-rich damaged-DNA compartments is most likely complicated and highly regulated, with a probable critical role of additional protein factors and phosphatases and/or kinases that remain to be identified. The hydrolysis of these compartments by PARG causes their dissociation and ensures reversibility of the whole repair process.

Thus, FUS may generate dynamic compartments in which damaged DNA accumulates, and this event should facilitate the recognition of DNA lesions by DNA repair proteins because of the relative increase in concentrations of damaged DNA and of repair factors within the compartments. The increase in the local concentration of DNA lesions and repair factors should accelerate DNA repair and its turnover in the cell.

## 5. Conclusions

Lately, a number of fundamental discoveries were made concerning the functions of RBPs in the DDR; they may profoundly change the concept of the regulation and organisation of DNA repair processes in the cell. Notably, the recent finding that RBPs with prion-like domains undergo phase separation points to the participation of RBPs in the formation of DNA damage–induced compartments [17,19,56,101,104]. In this regard, the interaction of RBPs with the PAR produced in response to DNA damage is currently receiving increasing attention because PAR not only contributes to the recruitment of RBPs to a DNA repair site but also promotes their LLPS. 

Since the discovery of the PAR-dependent recruitment of FUS to DNA repair foci in 2013 [51], there has been a substantial increase in the number of research articles confirming the role of FUS in DNA repair, thereby nurturing special interest in the question which molecular mechanisms may enable FUS to play a part in the DDR [33,34]. FUS seems to be involved in the DDR through an interaction with DNA repair intermediates, DNA repair factors and DNA damage signalling molecules like PAR [33,34]. Furthermore, FUS, having a self-adhesive LCD, can undergo phase separation [53,55,56,65,109,110]. Therefore, FUS–PAR interactions during DNA repair are possibly directly related to the assembly of damaged DNA with repair proteins and of transient repairosome compartments, which may carry out specific functions and implement spatiotemporal control over the DNA repair process [101]. To date, it is still unknown how FUS modulates DNA repair through compartmentalisation. Understanding the biological role of FUS in the generation of the repairosome and in its molecular composition and functions is necessary to clarify diverse characteristics of DNA repair–regulatory processes. 

Further research into FUS functions and into the other protein members of the FET family, EWSR1 and TAF15, may advance the present knowledge on the mechanisms of prion-like-RBP–dependent regulation of DNA repair processes through the formation of compartments in human cells. Future investigation of how these RBPs orchestrate DNA repair pathways will deepen our understanding of the response of the cancer cell to genotoxic stress and will elucidate the mutations in these proteins associated with neurological diseases as well as will lay the foundation for the development of relevant innovative preventive or therapeutic modalities. Notably, in neurodegenerative diseases, FUS forms cytoplasmic inclusions that can be toxic by themselves or may impair the DNA-related function of nuclear FUS in ALS or FTLD patients. Given that PARPs’ or PARG activities have been demonstrated to interfere with nucleocytoplasmic shuttling of FUS in the cytoplasm upon genotoxic stress [101,119], these data may extend the range of applications of PARPs or PARG inhibitors from cancer to neurodegenerative diseases [120].
